# Supplementary material for: Seminoma and Embryonal Carcinoma Footprints Identified by Analysis of Integrated Genome-Wide Epigenetic and Expression Profiles of Germ Cell Cancer Cell Lines
Source: PLoS One. 2014 Jun 2;9(6):e98330. doi: 10.1371/journal.pone.0098330 (PMC4041891; doi:10.1371/journal.pone.0098330)
Supplement: File S1 — ZIP file containing DMRforPairs output for significant regions. Please start from the html files. (ZIP) [file pone.0098330.s008.zip › figures/10467.pdf]

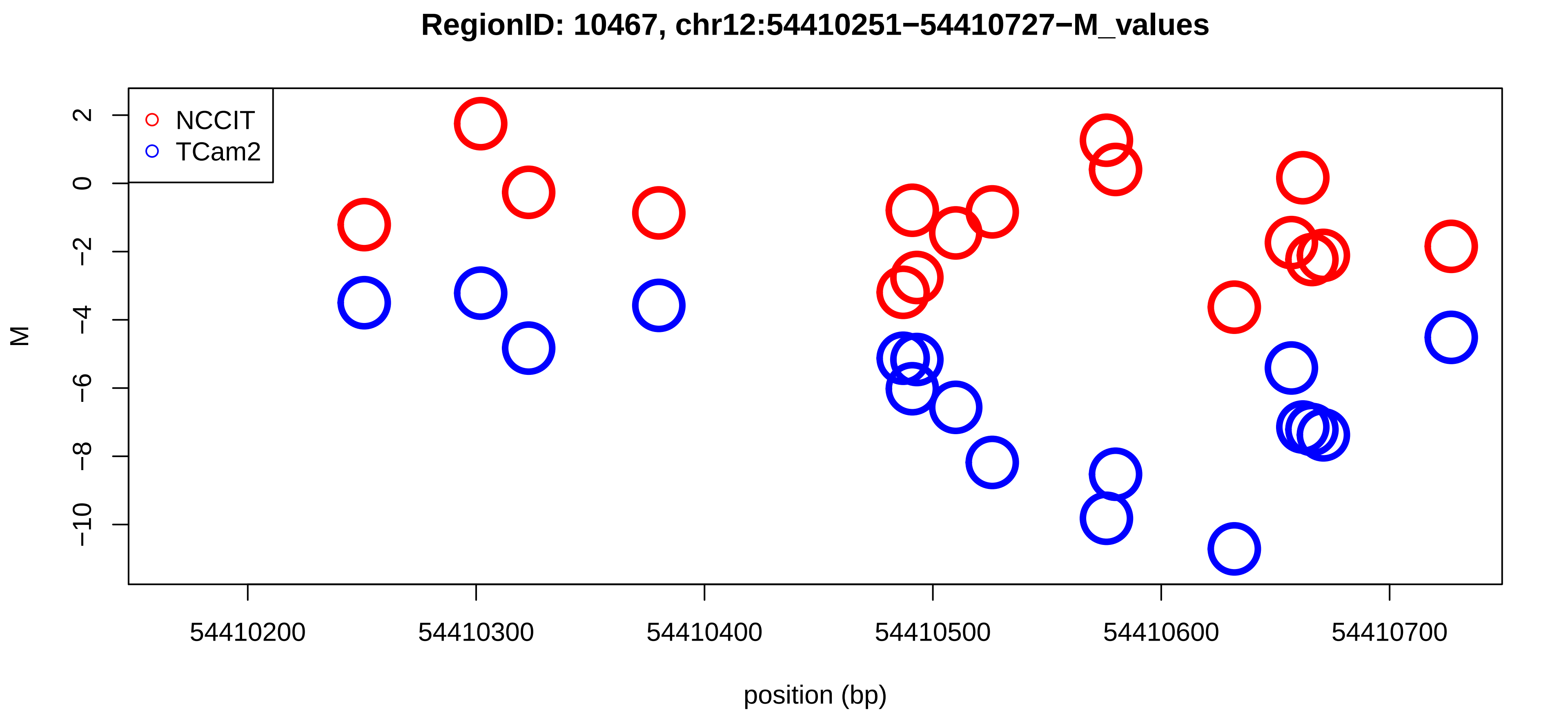

RegionID: 10467, chr12:54410251−54410727−M_values
NCCIT
TCam2
M
position (bp)

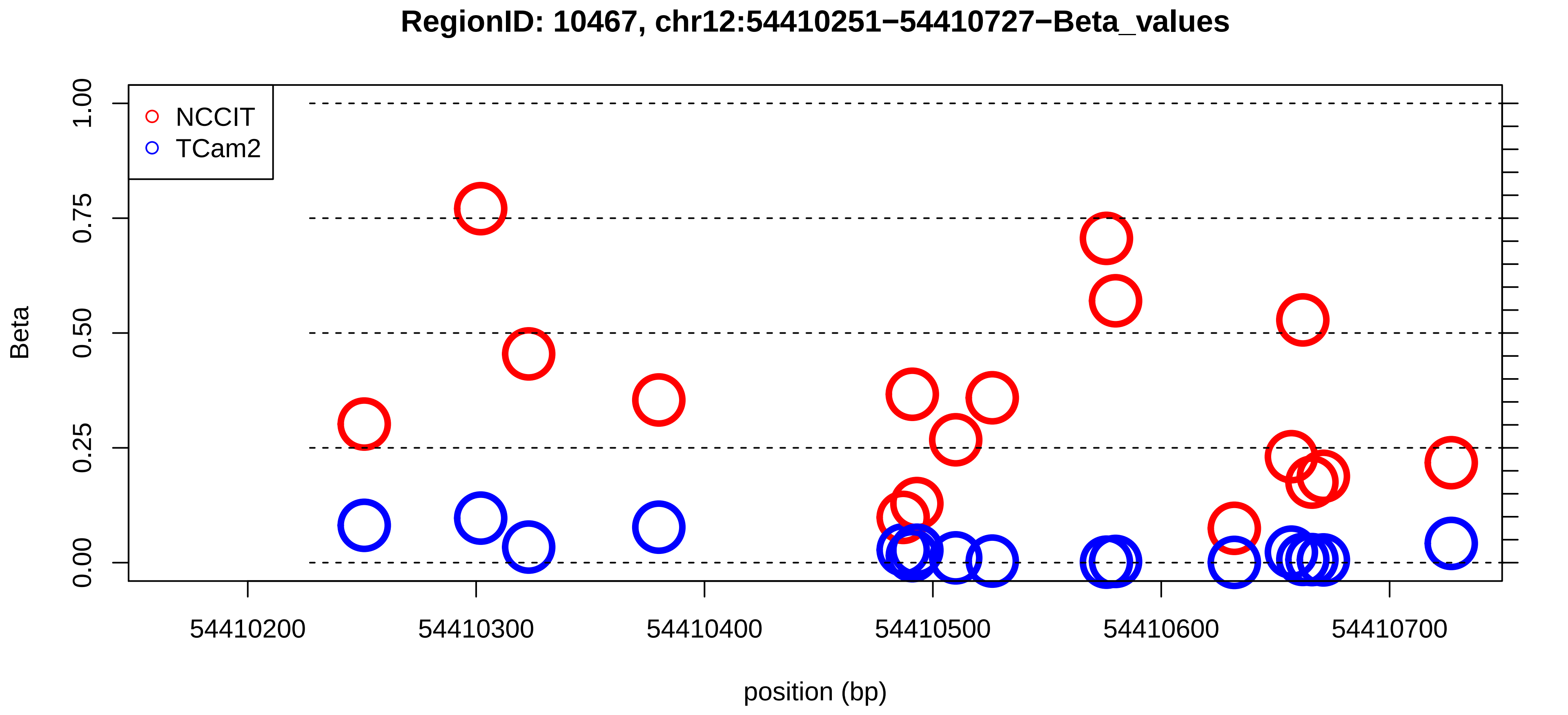

RegionID: 10467, chr12:54410251−54410727−Beta_values
NCCIT
TCam2
Beta
position (bp)

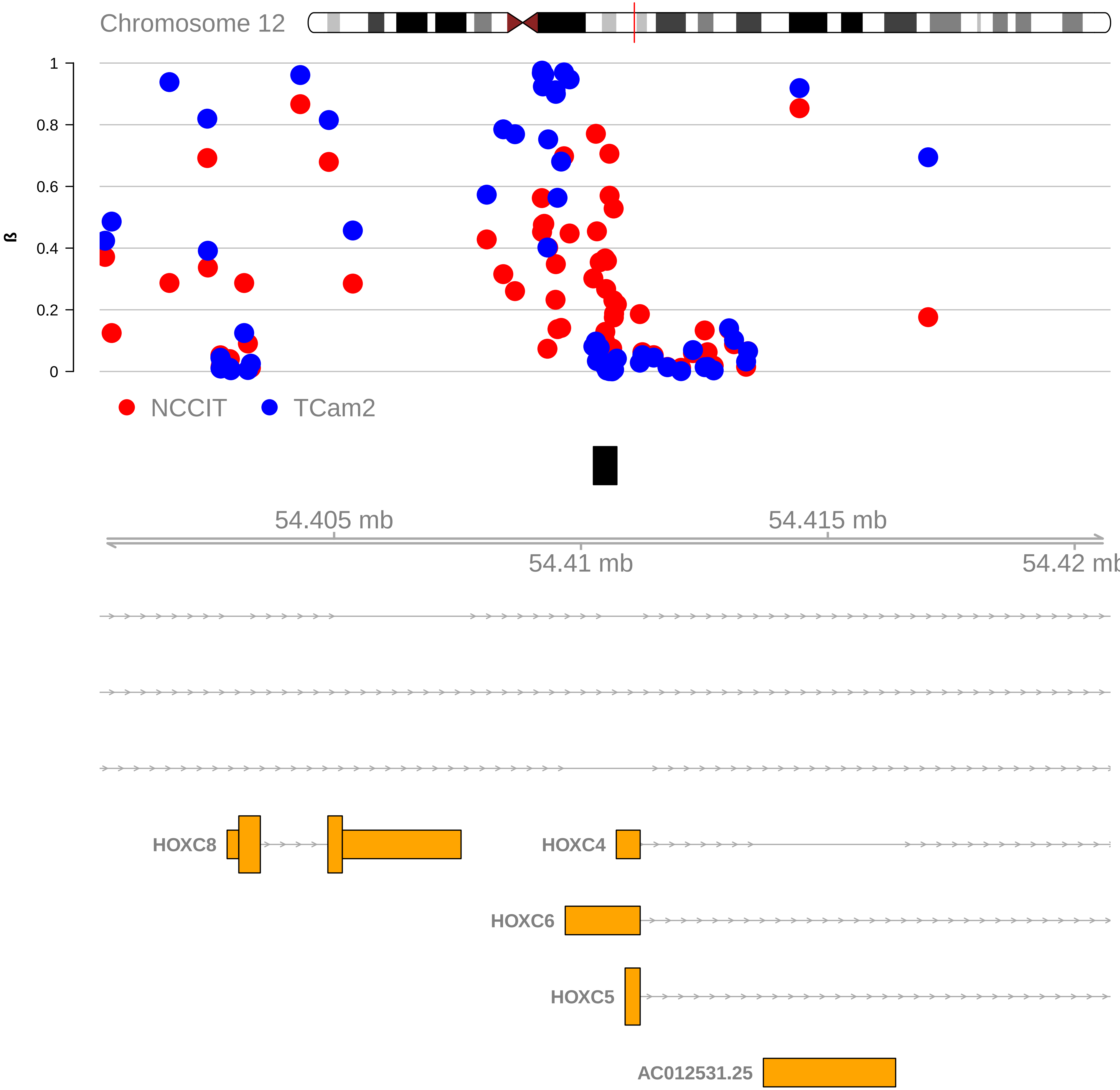

Chromosome 12
β
1
0.8
0.6
0.4
0.2
0
NCCIT
TCam2
54.405 mb
54.415 mb
54.41 mb
54.42 mb
HOXC8
HOXC4
HOXC6
HOXC5
AC012531.25
